# More effective strategies are required to strengthen public awareness of COVID-19: Evidence from Google Trends

**DOI:** 10.7189/jogh.10.011003

**Published:** 2020-06

**Authors:** Dingtao Hu, Xiaoqi Lou, Zhiwei Xu, Nana Meng, Qiaomei Xie, Man Zhang, Yanfeng Zou, Jiatao Liu, Guoping Sun, Fang Wang

**Affiliations:** 1 Department of Oncology, The First Affiliated Hospital of Anhui Medical University, Hefei, Anhui, China; 2 School of Public Health, Faculty of Medicine, University of Queensland. Queensland, Australia; 3 Department of Health Services Management, School of Health Management, Anhui Medical University, Hefei, Anhui, China; 4 Department of Epidemiology and Biostatistics, School of Public Health, Anhui Medical University, Hefei, Anhui, China; 5 Department of Pharmacy, The First Affiliated Hospital of Anhui Medical University, Hefei, Anhui, China

## Abstract

**Background:**

The outbreak of coronavirus disease 2019 (COVID-19) has posed stress on the health and well-being of both Chinese people and the public worldwide. Global public interest in this new issue largely reflects people’s attention to COVID-19 and their willingness to take precautionary actions. This study aimed to examine global public awareness of COVID-19 using Google Trends.

**Methods:**

Using Google Trends, we retrieved public query data for terms of “2019-nCoV + SARS-CoV-2 + novel coronavirus + new coronavirus + COVID-19 + Corona Virus Disease 2019” between the 31^st^ December 2019 and the 24^th^ February 2020 in six major English-speaking countries, including the USA, the UK, Canada, Ireland, Australia, and New Zealand. Dynamic series analysis demonstrates the overall change trend of relative search volume (RSV) for the topic on COVID-19. We compared the top-ranking related queries and sub-regions distribution of RSV about COVID-19 across different countries. The correlation between daily search volumes on the topic related to COVID-19 and the daily number of people infected with SARS-CoV-2 was analyzed.

**Results:**

The overall search trend of RSV regarding COVID-19 increased during the early period of observing time and reached the first apex on 31^st^ January 2020. A shorter response time and a longer duration of public attention to COVID-19 was observed in public from the USA, the UK, Australia, and Canada, than that in Ireland and New Zealand. A slightly positive correlation between daily RSV about COVID-19 and the daily number of confirmed cases was observed (*P* < 0.05). People across countries presented a various interest to the RSV on COVID-19, and public awareness of COVID-19 was different in various sub-regions within countries.

**Conclusions:**

The results suggest that public response time to COVID-19 was different across countries, and the overall duration of public attention was short. The current study reminds us that governments should strengthen the publicity of COVID-19 nationally, strengthen the public's vigilance and sensitivity to COVID-19, inform public the importance of protecting themselves with enough precautionary measures, and finally control the spread of COVID-19 globally.

The virus of the family Coronaviridae, which has been recognized in several hosts, including birds, as well as in some mammals, such as bats, camels, mice, dogs and cats, comprises a group of single-strand, positive-sense RNA viruses [1,2]. Previously, Coronaviruses (CoVs) were known to result in several clinical symptoms in humans like colds and diarrhea [3]. Since most of the illnesses caused by coronavirus are mild, people did not pay great attention to these viruses. However, there are two notable exceptions: Middle East Respiratory Syndrome (MERS) coronavirus (MERS-CoV) and Severe Acute Respiratory Syndrome (SARS) coronavirus (SARS-CoV), which resulted in 858 fatalities and 774 deaths respectively [4,5]. Moreover, it is reported that SARS-CoV was mainly found in bats, and people generally believed that this virus might cause another disease outbreak in the future [6].

In late December 2019, several cases of severe pneumonia of unknown coronavirus infected at the local Huanan seafood wholesale market in Wuhan (China) were reported [7]. The seafood market was found to sold seafood and several live wild animals, including marmots, bats and other mammals, which suggests there might be a potential zoonotic origin. The cluster cases of pneumonia of unknown cause detected in Wuhan, China was reported to the World Health Organization (WHO) on the 31^st^ December 2019, and this new virus was named 2019 novel coronavirus (2019-nCoV) temporarily [8]. Officially, this novel coronavirus was named severe acute respiratory syndrome coronavirus 2 (SARS-CoV-2), by the International Committee on Taxonomy of Virus (ICTV) on the 11^th^ February 2020, and the diseases caused by SARS-CoV-2 were named Corona Virus Diseases 2019 (COVID-19) on the same day. As a cousin of SARS-CoV, SARS-CoV-2 is identified with lighter symptoms and stronger transmissibility. As of the 28^th^ February 2020, more than 79 000 cases were reported in mainland China, including 2835 deaths [9]. Besides China, cases confirmed with COVID-19 had also been detected in many other countries and territories, including Japan, Malaysia, Singapore, and the USA [10]. Given the dreadful situation, China had quarantined 35 million people in Wuhan and some other cities, try to limit the spread of the virus [11]. Other countries also took action. The ports of entry in many countries were travel screened particularly for people from China[12]. Yet, with the case number soaring, these actions may be too late. Global public interest in COVID-19 largely reflects people’s attention and their willingness to take precautionary actions like staying at home, avoid going out and gathering, wearing masks, and avoid contacting with anyone with the symptoms of cold or influenza. There are already many studies carried on COVID-19, however, few focused on the public interest in this issue [11-14]. Our research aimed to fill that void.

Over the past two decades, with Internet availability and usage increased worldwide, people get information mainly through this novel alternative method [15]. Google, in particular, as the most popular search engine, provides a web site called Google Trends, which analysis the popularity of specific search terms in this novel query engine worldwide [16]. Since Google Trends became available to the public, it has been implemented to examine several time-ranking patterns of some health-related issues to investigate public awareness of these diseases, including mental illnesses [17], gout [18], and cellulitis [19].

Therefore, we aimed to explore public awareness of COVID-19 by the query data retrieved from Google Trends in this research.

## METHODS

### Search terms selection and data collection

There is a publicly available web-based tool, which provides the number of relative searches within a certain region or globally for a particular query, known as Google Trends (support.google.com/trends). To make comparisons between queries more accurate, the data retrieved from Google Trends is normalized against total search volume, and the repetitive searches conducted by the same user in a short time from is automatically eliminated. Rather than provide the absolute, row search figures for statistical analyses, Google Trends present the relative search volume (RSV) instead. The data collected from Google Trends is adjusted to the time and location, so the comparisons between queries can be easier. The results can be downloaded in the format of Common Separated Values, which displayed on a scale from 0 to 100. Before the analysis, two separate individuals had cross-checked the data to access the accuracy.

Since the officially names of “SARS-CoV-2” and “COVID-19” were defined on the 11^th^ February 2020, 43 days after the day China reported to the WHO. To better analysis public awareness of this issue, we use the following search terms “2019-nCoV” “SARS-CoV-2”, “novel coronavirus”, “new coronavirus”, “COVID-19” and “Corona Virus Disease 2019” (which were most searched) to investigate public awareness of COVID-19 in six major English-speaking countries from both hemispheres. Additionally, plus sign (+) which means “OR” was used to represent the combination of those multiple terms. These queries were searched from 2019/12/31 to 2020/02/24 within the countries from the Northern hemisphere, including the USA, the UK, Canada and Ireland as well as countries from the Southern hemisphere, including Australia and New Zealand. These countries were selected because they reflect the major native English-speaking countries in both hemispheres, and the searches were conducted on the 28^th^ February 2020. For the purpose of comparing the difference of public response to the topic about COVID-19 across countries, we defined two novel definitions to make the comparisons easier: response time and duration of public attention. Response time refers to the time between the day when WHO received the report, and to the first day when the RSV started to occur. While the duration of public attention refers to the time between the day when RSV started to occur to the day it reached the apex (time points at both ends were included in response time and duration of public attention). To analysis the difference between the Northern and Southern hemisphere, we get the data which represent the RSV related to COVID-19 in the Northern hemisphere by adding up the daily RSV in the USA, the UK, Canada and Ireland. Using the same way, we get the data in the Southern hemisphere by combining the daily RSV in Australia and New Zealand. Besides, since the RSV in the United States is less than “1” for several days, we treated it as RSV equal to “1” in these days for better reflecting the American response to COVID-19.

### Dynamic series analysis

Arrange a series of statistical indicators in chronological order, you get the dynamic series analysis, which can be used to illustrate the development and change trend of a particular object in time. In this study, the dynamic series analysis provides several statistic indicators, including the daily development and increment rate as well as the fixed base ratio and link-relative ratio.

The daily development rate of fixed base refers to the relative number obtained by comparing the RSV in a given period with the baseline, which reflects the general development direction of RSV in a certain period of time. While the development of link-relative refers to the ratio between the certain day’s RSV and the search volume of the previous time, which signals the day by day growth rate of the relative search volume. Since the cardinal number cannot be zero, we take the first day when RSV is not zeroing as the baseline. In addition, you can get the increment rate by decreasing the development rate by 100%. Besides, visual inspections for our results were presented by software Graphpad Prism 8.2 (Graphpad Inc, Harvey Motulsky, USA) and ArcGIS 10.6 (ESRI Inc, Jack Dangermond, Redlands, CA, USA).

### Statistical analysis

We use SPSS version 24.0 (IBM Inc, Armonk, NY, USA) to conduct a correlation analysis between the daily search volumes of “2019-nCoV + SARS-CoV-2 + novel coronavirus + new coronavirus + COVID-19 + Corona Virus Disease 2019” and the daily number of confirmed cases with COVID-19 pneumonia. Since the data are not normally distributed, we pick the Spearman correlation instead of Pearson correlation to investigate our data [20]. *P* < 0.05 indicates that there is a statistically significant correlation between variables.

### Ethics considerations

The search query trend data gathered from Google trends was freely available information and fully anonymized. In the health care section, we performed this research according to the Helsinki declaration and adhered to the recommendation of a current overview on Google trends.

## RESULTS

### Overall search trend of the RSV for the topic regarding COVID-19

**Figure 1:**
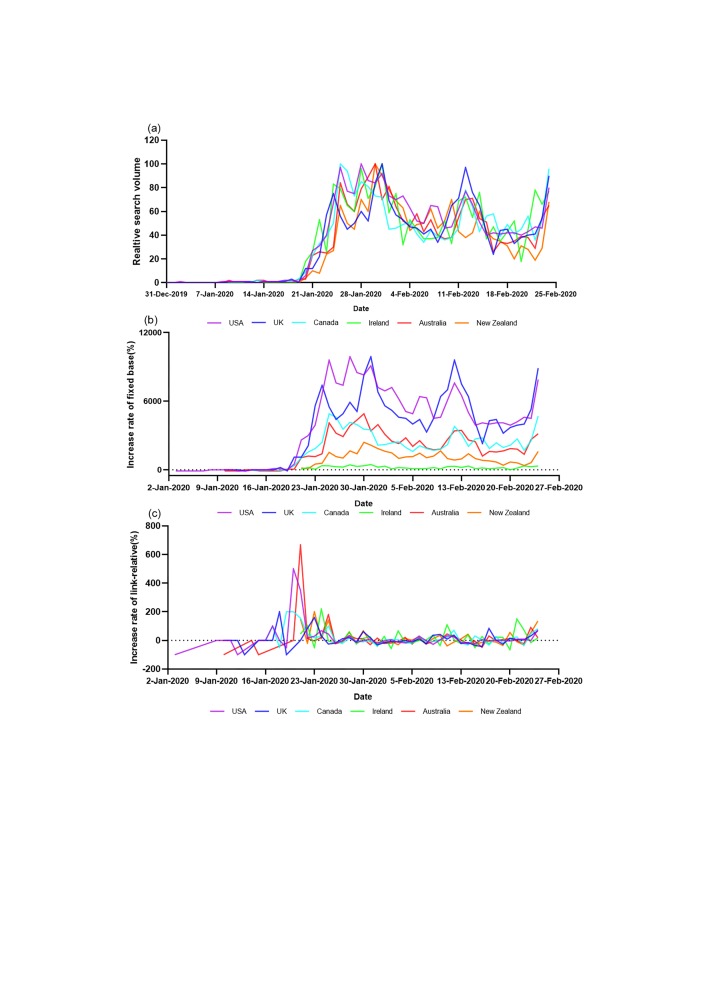
Relative search volume (RSV) and the increase rate of RSV regarding COVID-19 in 6 mainly English-speaking countries (a, b and c represent the overall change trend of RSV, the ratio of fixed base and link-relative, respectively).

The overall search trend of “2019-nCoV + SARS-CoV-2 + novel coronavirus + new coronavirus + COVID-19 + Corona Virus Disease 2019” increased during the early period of observing time and reached a summit around the 31^st^ January 2020, then the trend declined. The second apex occurred near the 12^th^ February 2020 and decreased again until the 19^th^ February 2020. After that, the overall trend keeps increasing. The first peak value occurred around the 31^st^ January 2020 in most countries, including the UK, Ireland, Australia and New Zealand. The USA and Canada as an exception, the first summit of RSV in these countries occurred on the 28^th^ January 2020 and the 25^th^ January 2020, respectively, earlier than the other four countries. (**Table 1**, **Figure 1**, Table S1 in the **Online Supplementary Document**).

**Table 1:** Dynamic series analysis of the search volumes for the topic regarding COVID-19 in the USA and the UK

Country	Date	RSV	Absolute increment		Development rate(%)		Increment rate(%)	
**Cumulative**	**Day on day**	**Fixed base ratio**	**Link relative ratio**	**Fixed base ratio**	**Link relative ratio**
USA	02-Jan-20	1	–	–	100.0	100.0	–	–
	03-Jan-20	0	-1	-1	0.0	0.0	-100.0	-100.0
	04-Jan-20	0	-1	0	0.0	–	-100.0	–
	05-Jan-20	0	-1	0	0.0	–	-100.0	–
	06-Jan-20	0	-1	0	0.0	–	-100.0	–
	07-Jan-20	0	-1	0	0.0	–	-100.0	–
	08-Jan-20	1	0	1	100.0	–	0.0	–
	09-Jan-20	1	0	0	100.0	100.0	0.0	0.0
	10-Jan-20	1	0	0	100.0	100.0	0.0	0.0
	11-Jan-20	1	0	0	100.0	100.0	0.0	0.0
	12-Jan-20	0	-1	-1	0.0	0.0	-100.0	-100.0
	13-Jan-20	0	-1	0	0.0	–	-100.0	_
	14-Jan-20	1	0	1	100.0	_	0.0	_
	15-Jan-20	1	0	0	100.0	100.0	0.0	0.0
	16-Jan-20	1	0	0	100.0	100.0	0.0	0.0
	17-Jan-20	2	1	1	200.0	200.0	100.0	100.0
	18-Jan-20	2	1	0	200.0	100.0	100.0	0.0
	19-Jan-20	1	0	-1	100.0	50.0	0.0	-50.0
	20-Jan-20	6	5	5	600.0	600.0	500.0	500.0
	21-Jan-20	27	26	21	2700.0	450.0	2600.0	350.0
	22-Jan-20	31	30	4	3100.0	114.8	3000.0	14.8
	23-Jan-20	40	39	9	4000.0	129.0	3900.0	29.0
	24-Jan-20	67	66	27	6700.0	167.5	6600.0	67.5
	25-Jan-20	97	96	30	9700.0	144.8	9600.0	44.8
	26-Jan-20	77	76	-20	7700.0	79.4	7600.0	-20.6
	27-Jan-20	75	74	-2	7500.0	97.4	7400.0	-2.6
	28-Jan-20	100	99	25	10 000.0	133.3	9900.0	33.3
	29-Jan-20	86	85	-14	8600.0	86.0	8500.0	-14.0
	30-Jan-20	84	83	-2	8400.0	97.7	8300.0	-2.3
	31-Jan-20	92	91	8	9200.0	109.5	9100.0	9.5
	01-Feb-20	73	72	-19	7300.0	79.3	7200.0	-20.7
	02-Feb-20	70	69	-3	7000.0	95.9	6900.0	-4.1
	03-Feb-20	73	72	3	7300.0	104.3	7200.0	4.3
	04-Feb-20	63	62	-10	6300.0	86.3	6200.0	-13.7
	05-Feb-20	52	51	-11	5200.0	82.5	5100.0	-17.5
	06-Feb-20	50	49	-2	5000.0	96.2	4900.0	-3.8
	07-Feb-20	65	64	15	6500.0	130.0	6400.0	30.0
	08-Feb-20	64	63	-1	6400.0	98.5	6300.0	-1.5
	09-Feb-20	46	45	-18	4600.0	71.9	4500.0	-28.1
	10-Feb-20	47	46	1	4700.0	102.2	4600.0	2.2
	11-Feb-20	62	61	15	6200.0	131.9	6100.0	31.9
	12-Feb-20	77	76	15	7700.0	124.2	7600.0	24.2
	13-Feb-20	66	65	-11	6600.0	85.7	6500.0	-14.3
	14-Feb-20	51	50	-15	5100.0	77.3	5000.0	-22.7
	15-Feb-20	40	39	-11	4000.0	78.4	3900.0	-21.6
	16-Feb-20	42	41	2	4200.0	105.0	4100.0	5.0
	17-Feb-20	41	40	-1	4100.0	97.6	4000.0	-2.4
	18-Feb-20	42	41	1	4200.0	102.4	4100.0	2.4
	19-Feb-20	42	41	0	4200.0	100.0	4100.0	0.0
	20-Feb-20	40	39	-2	4000.0	95.2	3900.0	-4.8
	21-Feb-20	43	42	3	4300.0	107.5	4200.0	7.5
	22-Feb-20	47	46	4	4700.0	109.3	4600.0	9.3
	23-Feb-20	46	45	-1	4600.0	97.9	4500.0	-2.1
	24-Feb-20	80	79	34	8000.0	173.9	7900.0	73.9
UK	09-Jan-20	1	–	–	100.0	100.0	–	–
	10-Jan-20	1	0	0	100.0	100.0	0.0	0.0
	11-Jan-20	1	0	0	100.0	100.0	0.0	0.0
	12-Jan-20	1	0	0	100.0	100.0	0.0	0.0
	13-Jan-20	0	-1	-1	0.0	0.0	-100.0	-100.0
	14-Jan-20	1	0	1	100.0	–	0.0	–
	15-Jan-20	1	0	0	100.0	100.0	0.0	0.0
	16-Jan-20	1	0	0	100.0	100.0	0.0	0.0
	17-Jan-20	1	0	0	100.0	100.0	0.0	0.0
	18-Jan-20	3	2	2	300.0	300.0	200.0	200.0
	19-Jan-20	0	-1	-3	0.0	0.0	-100.0	-100.0
	20-Jan-20	12	11	12	1200.0	–	1100.0	–
	21-Jan-20	12	11	0	1200.0	100.0	1100.0	0.0
	22-Jan-20	22	21	10	2200.0	183.3	2100.0	83.3
	23-Jan-20	57	56	35	5700.0	259.1	5600.0	159.1
	24-Jan-20	75	74	18	7500.0	131.6	7400.0	31.6
	25-Jan-20	56	55	-19	5600.0	74.7	5500.0	-25.3
	26-Jan-20	45	44	-11	4500.0	80.4	4400.0	-19.6
	27-Jan-20	50	49	5	5000.0	111.1	4900.0	11.1
	28-Jan-20	60	59	10	6000.0	120.0	5900.0	20.0
	29-Jan-20	52	51	-8	5200.0	86.7	5100.0	-13.3
	30-Jan-20	84	83	32	8400.0	161.5	8300.0	61.5
	31-Jan-20	100	99	16	10 000.0	119.0	9900.0	19.0
	01-Feb-20	69	68	-31	6900.0	69.0	6800.0	-31.0
	02-Feb-20	57	56	-12	5700.0	82.6	5600.0	-17.4
	03-Feb-20	53	52	-4	5300.0	93.0	5200.0	-7.0
	04-Feb-20	47	46	-6	4700.0	88.7	4600.0	-11.3
	05-Feb-20	46	45	-1	4600.0	97.9	4500.0	-2.1
	06-Feb-20	41	40	-5	4100.0	89.1	4000.0	-10.9
	07-Feb-20	45	44	4	4500.0	109.8	4400.0	9.8
	08-Feb-20	34	33	-11	3400.0	75.6	3300.0	-24.4
	09-Feb-20	46	45	12	4600.0	135.3	4500.0	35.3
	10-Feb-20	65	64	19	6500.0	141.3	6400.0	41.3
	11-Feb-20	71	70	6	7100.0	109.2	7000.0	9.2
	12-Feb-20	97	96	26	9700.0	136.6	9600.0	36.6
	13-Feb-20	76	75	-21	7600.0	78.4	7500.0	-21.6
	14-Feb-20	65	64	-11	6500.0	85.5	6400.0	-14.5
	15-Feb-20	42	41	-23	4200.0	64.6	4100.0	-35.4
	16-Feb-20	24	23	-18	2400.0	57.1	2300.0	-42.9
	17-Feb-20	44	43	20	4400.0	183.3	4300.0	83.3
	18-Feb-20	45	44	1	4500.0	102.3	4400.0	2.3
	19-Feb-20	33	32	-12	3300.0	73.3	3200.0	-26.7
	20-Feb-20	38	37	5	3800.0	115.2	3700.0	15.2
	21-Feb-20	40	39	2	4000.0	105.3	3900.0	5.3
	22-Feb-20	41	40	1	4100.0	102.5	4000.0	2.5
	23-Feb-20	54	53	13	5400.0	131.7	5300.0	31.7
	24-Feb-20	90	89	36	9000.0	166.7	8900.0	66.7

RSV – relative search volume

### Increase rate of fixed base and link-relative in different countries

Public response speed toward the topic regarding COVID-19 was different in various countries. The highest link-relative ratio, which reflects the highest rate of RSV increment, occurred around the 20^th^ January 2020 in most countries contained in current research, including the United States, the UK, Canada and Australia. However, the highest rate of link-relative in Ireland (the 24^th^ January 2020) and New Zealand (the 23^rd^ January 2020) appeared later than that in the USA (the 20^th^ January 2020), the UK (the 18^th^ January 2020), Canada (the 20^th^ January 2020) and Australia (the 21^st^ January 2020). Of interest, the overall value of the fixed base ratio in Ireland and New Zealand was smaller than that in the other four countries contained in our study. This indicates the fluctuation of RSV for the topic regarding COVID-19 was lighter than other countries (**Table 1**, **Figure 1**, Table S1 in the **Online Supplementary Document**).

### Response time and duration of public attention

**Figure 2:**
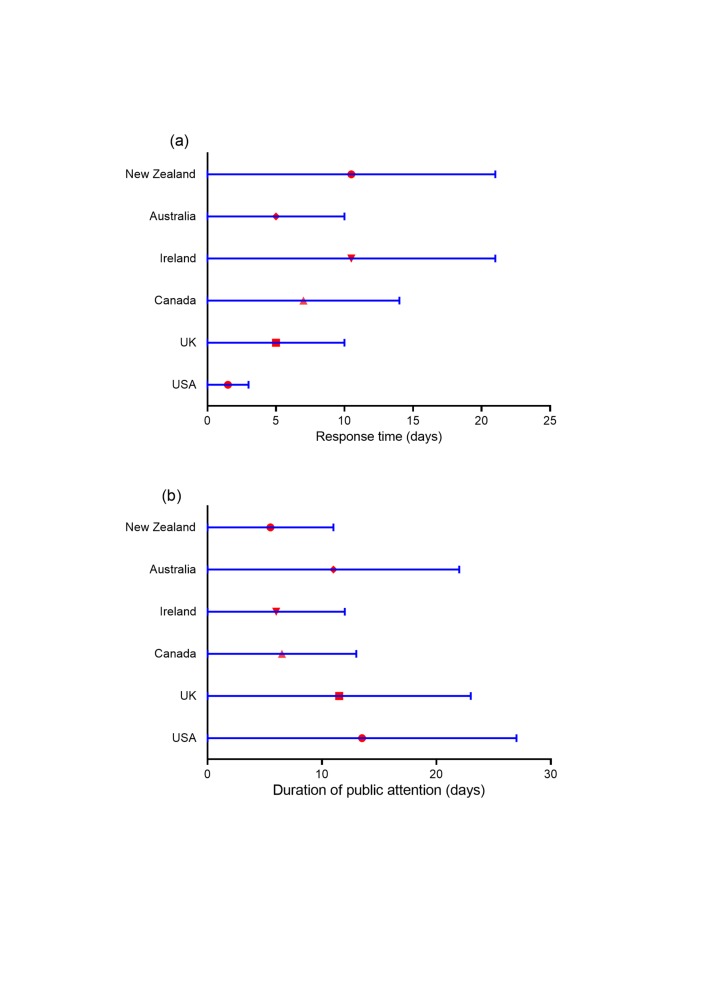
Response time (a) and duration of public attention (b) toward COVID-19 in 6 major English-speaking countries.

The response time and the duration of public attention were different in various countries. The response time in the USA (from the 31^st^ December 2019 to 2^nd^ January 2020), the UK (from the 31^st^ December 2019 to 9^th^ January 2020), Australia (from the 31^st^ December 2019 to the 9^th^ January 2020) and Canada (from the 31^st^ December 2019 to the 13^th^ January 2020) was 3 days, 10 days, 10 days, and 14 days, respectively. However, the time of response in Ireland (from the 31^st^ December 2019 to the 20^th^ January 2020) and New Zealand (from the 31^st^ December 2019 to the 20^th^ January 2020) was 21 days, much too longer than that in other four countries. Which indicates the public in Ireland and New Zealand had a slower response speed toward COVID-19. Also, the duration of public attention in the USA (from the 2^nd^ January 2020 to the 28^th^ January 2020, 27 days), the UK (from the 9^th^ January 2020 to the 31^st^ January 2020, 23 days), Australia (from the 9^th^ January 2020 to the 30^th^ January 2020, 22 days), and Canada (from the 13^th^ January 2020 to the 25^th^ January 2020, 13 days) was longer than that in Ireland (from the 20^th^ January 2020 to the 31^st^ January 2020, 12 days), and New Zealand (from the 20^th^ January 2020 to the 30^th^ January 2020, 11 days) (**Table 2**, **Figure 2**). Besides, the median time of response in the countries contained in our study was 12 days, while the median time of duration of public attention was 17.5 days.

**Table 2:** Response time and duration of public attention to COVID-19 in major English-speaking countries

Countries	Response time			Duration of public attention			
**China reported to WHO**	**Start to response**	**Total (days)**	**Start to response**	**Apex**	**Total (days)**	
USA	31-Dec-19	02-Jan-20	3	02-Jan-20	28-Jan-20		27
UK	31-Dec-19	09-Jan-20	10	09-Jan-20	31-Jan-20		23
Canada	31-Dec-19	13-Jan-20	14	13-Jan-20	25-Jan-20		13
Ireland	31-Dec-19	20-Jan-20	21	20-Jan-20	31-Jan-20		12
Australia	31-Dec-19	09-Jan-20	10	09-Jan-20	30-Jan-20		22
New Zealand	31-Dec-19	20-Jan-20	21	20-Jan-20	30-Jan-20		11

WHO – World Health Organization

### Correlation analysis for the RSV related to COVID-19 with the daily number of confirmed cases in China and the total daily number of confirmed patients in other countries (besides China)

The correlation between the RSV for “2019-nCoV + SARS-CoV-2 + novel coronavirus + new coronavirus + COVID-19 + Corona Virus Disease 2019” and number of confirmed cases was revealed by Spearman correlation analysis (Table S2, Figures S1 and S2 in the **Online Supplementary Document**). Results showed the relative search volume for the topic of COVID-19 was slight positively correlated with the number of Chinese confirmed cases in the USA (r_s_ = 0.416, *P* = 0.004), the UK (r_s_ = 0.495, *P* = 4.727 × 10^-4^), Canada (r_s_ = 0.540, *P* = 1.090 × 10^-4^), Ireland (r_s_ = 0.490, *P* = 0.001), Australia (r_s_ = 0.483, *P* = 0.001), New Zealand (r_s_ = 0.463, *P* = 0.001), as well as the Northern hemisphere (r_s_ = 0.482, *P* = 0.001) and the Southern hemisphere (r_s_ = 0.459, *P* = 0.001). Similar results also found in the correlation between RSV for “2019-nCoV + SARS-CoV-2 + novel coronavirus + new coronavirus + COVID-19 + Corona Virus Disease 2019” and total daily number of confirmed cases in other countries (except China) (UK: r_s_ = 0.388, *P* = 0.01; Canada: r_s_ = 0.439, *P* = 0.003; Ireland: r_s_ = 0.380, *P* = 0.012; Australia: r_s_ = 0.372, *P* = 0.014; New Zealand: r_s_ = 0.348, *P* = 0.022; the Northern hemisphere: r_s_ = 0.368, *P* = 0.015 and the Southern hemisphere: r_s_ = 0.343, *P* = 0.024). However, the correlation was not strong in both analyses. Besides, no correlation was found between the RSV regarding COVID-19 in the USA (*P* = 0.061) and the total daily number of cases in other countries (except China).

### Relative growth of topic regarding COVID-19 in major English-speaking countries

Results of the analysis for the relative search terms about COVID-19 were presented in Table 3 (USA: “coronavirus new york”, “is coronavirus new”, “coronavirus china”, “new virus”, “coronavirus in new york”; UK: “coronavirus uk”, “coronavirus new york”, “coronavirus symptoms”, “new zealand coronavirus”, “coronavirus china”; Canada: “coronavirus Canada”, “coronavirus china”, “coronavirus symptoms”, “lunar new year”, “coronavirus new name”; Australia: “new zealand coronavirus”, “coronavirus symptoms”, “novel coronavirus australia”, “coronavirus update”, “novel coronavirus 2019”; New Zealand: “new zealand coronavirus”, “coronavirus nz”, “coronavirus in new Zealand”, “is coronavirus in new Zealand”, “is the coronavirus in new zealand”), arranged from high to low according to the growth of search queries related to the COVID-19. Ireland was excluded since Google Trends did not generate enough data from this country to investigate the relative search terms regarding COVID-19 and comparisons of RSV within regions. The results suggest that people across countries did not only care about the transmission of COVID-19 in their own countries, but also in other countries. Besides, people in the UK, Canada and Australia also cared about the symptoms of COVID-19, and public interest in the topic “lunar new year” was observed in Canada.

**Table 3:** Top ranking relative queries and sub-regions regarding COVID-19 in the USA, the UK, Canada, Australia and New Zealand

Countries	Rank	Related topic	RSV	Sub-regions	RSV
USA	1	coronavirus new york	100	New York	100
	2	is coronavirus new	40	New Jersey	83
	3	coronavirus china	38	New Mexico	80
	4	new virus	35	District of Colombia	72
	5	coronavirus in new york	30	New Hampshire	69
UK	1	coronavirus uk	100	England	100
	2	coronavirus new york	58	Northern Ireland	90
	3	coronavirus symptoms	56	Wales	87
	4	new zealand coronavirus	54	Scotland	82
	5	coronavirus china	50	_	-
Canada	1	coronavirus canada	100	British Columbia	100
	2	coronavirus china	55	Ontario	72
	3	coronavirus symptoms	39	Nova Scotia	65
	4	lunar new year	35	Alberta	63
	5	coronavirus new name	27	New Brunswick	59
Australia	1	new zealand coronavirus	100	Australia Capital Territory	100
	2	coronavirus symptoms	90	Northern Territory	69
	3	novel coronavirus australia	47	South Australia	62
	4	coronavirus update	36	New South Wales	62
	5	novel coronavirus 2019	36	Victoria	59
New Zealnd	1	new zealand coronavirus	100	West Coast	100
	2	coronavirus nz	28	Auckland	81
	3	coronavirus in new zealand	22	Taranaki	81
	4	is coronavirus in new zealand	7	Southland	74
	5	is the coronavirus in new zealand	6	Otago	65

RSV – relative search volumes

### Top ranking sub-regions distribution of RSV for the topic regarding COVID-19 in different countries

**Figure 3:**
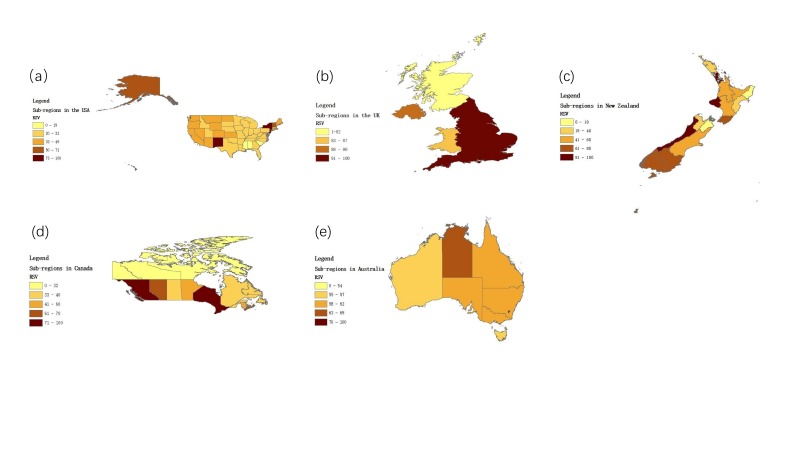
Graphic map of search term popularity for “2019-nCoV + SARS-CoV-2 + novel coronavirus + new coronavirus + COVID-19 + Corona Virus Disease 2019” by location.

Google’s outputs for data analysis in different countries and sub-regions were demonstrated in **Table 3** and **Figure 3**. Top 5 ranking sub-regions with a higher RSV regarding COVID-19 were New York, New Jersey, New Mexico, District of Colombia, New Hampshire, (in the USA); England, Northern Ireland, Wales, Scotland (in the UK); British Columbia, Ontario, Nova Scotia, Alberta, New Brunswick (in Canada); Australia Capital Territory, Northern Territory, South Australia, New South Wales, Victoria (in Australia); West Coast, Auckland, Taranaki, Southland, Otago, (in New Zealand). For the same reason, we did not include Ireland in this analysis. Moreover, People in Northwest Territories, Nunavut, and Yukon, places in the northwest of Canada, did not present too much attention to the topic regarding COVID-19 (**Figure 3**).

## DISCUSSION

Data collected from six mainly English-speaking countries by Google Trends indicate that public response speed toward COVID-19 was different across countries. The overall search trend for the RSV regarding COVID-19 increased in the early period of observing time, reached an apex around the 31^st^ January 2020 in most countries contained in our study. The highest increment speed in New Zealand and Ireland occurred later than that in the United States, the UK, Canada and Australia. In addition, people in the USA, the UK, Australia, and Canada had a faster response speed than that in Ireland and New Zealand. Moreover, the duration of public attention in the USA, the UK, Australia and Canada was longer than that in Ireland and New Zealand. Our results also suggest that RSV for the topic of COVID-19 in most countries was slightly positively correlated with the number of patients infected with SARS-CoV-2 in China and the cases confirmed with COVID-19 in other countries (besides China). In addition, people from different countries presented various interests to the RSV related to COVID-19, and people’s attention to COVID-19 was also different in various sub-regions within each country. Some reasons may contribute to our results.

In late December 2019, some patients were reported because of an unidentified virus in Wuhan, China, presenting with the symptoms of severe pneumonia. However, other countries did not pay much attention to SARS-CoV-2 until the National Health Commission of China has listed SARS-CoV-2 as class B infectious disease and treated as Class A for infection prevention and control management on th 20^th^ January 2020 [9]. This indicates this novel coronavirus can be a severe threat to public health. This may be a reason that the search trends about COVID-19 reached the highest increment speed near the 20^th^ January 2020. Besides, the first confirmed case with SARS-CoV-2 infection reported by the WHO in the USA, and Australia is on the 21^st^ January 2020 and the 25^th^ January 2020, respectively [9]. Coincidentally, the day when the second-highest increment speed occurred is exactly the day when the first infected case was reported. Also, the first peak value of the RSV regarding COVID-19 in Canada occurred on the 25^th^ January 2020, while the first confirmed case was also reported on the same day. According to the WHO’s report on the 30^th^ January 2020 that the outbreak of COVID-19 meets the criteria for a Public Health Emergency of International Concern (PHEC). Which indicates that this epidemic may not only post a severe threat to the health and well-being of Chinese people, but may have also posed stress on the economy of the whole world. So, the summit of RSV occurred around the 31^st^ January 2020 in most countries from both hemispheres. The peak value of RSV regarding COVID-19 in the UK occurred on the 31^st^ January 2020 may also because of the first confirmed case within the UK was reported on that day. Also, until the 24^th^ February 2020, there were no confirmed cases had been reported in Ireland and New Zealand, which may contribute to the highest increment speed in Ireland and New Zealand occurred later than the other four countries. Besides, the response time in Ireland and New Zealand was also longer than that in other countries, which indicates the public in these countries had a lower vigilance to COVID-19 compared with people in the USA, the UK, Australia, and Canada. The official names of the novel coronavirus and diseases caused by this virus were confirmed on th e11^th^ Feb 2020, which may lead to the emergence of the second apex for RSV about COVID-19. Besides, the first cluster of passengers detected negatively of SARS-CoV-2, began leaving the Diamond Princess and back to their own countries on th e19^th ^February 2020. Thus, people’s interest in COVID-19 increased again from the 19^th^ February 2020, and with the case number soaring worldwide, the search trend keeps increasing.

Besides, people in the USA, the UK, Australia, and Canada had a faster response speed toward COVID-19 than other countries contained in current research, and the duration of public attention was longer than that in Ireland and New Zealand as well. The health care system within the United States plays a critical role in enhancing public awareness toward health-related issues. Since the outbreak of COVID-19, Centers for Disease Control and Prevention (CDC), multiple other federal and local agencies, as well as other partners and health departments have implemented robust methods to prevent the further spread of COVID-19 in the USA [21,22]. Besides, according to the statistics from the WHO and CDC that up to more than 19 million people have been diagnosed with influenza in America, with more than 10 thousand fatalities as of the 25^th^ January 2020. So, the public in America may be more sensitive to the topic regarding COVID-19 than in other countries. According to Matt Hancock, England’s health secretary, that England has a world-leading infection control procedure [23]. Before the first confirmed cases had been found in England, Britain had done enough “self-isolation” measures, in case of the possible transmission of COVID-19 in the UK. According to the Foreign and Commonwealth Office (FCO)’s plan for the evacuation of overseas Chinese, 83 British citizens in Wuhan had flown back to the UK on January 31 and been isolated for 14 days at a military base in Oxfordshire, UK [24]. Which confirmed that the infection-control procedures in the UK are advanced. Besides, Australia had also implemented many measures, including denied the people who had left or transited through mainland China gets into Australia, to slow the spread of COVID-19 into Australia and to prepare health care laboratories and services for a timely response [25]. However, as of the 24^th^ February 2020, the number of patients confirmed with COVID-19 in Canada (10 cases) was lower than in the USA (35 cases), the UK (13 cases) and Australia (22 cases) [9]. This may be an explanation of why the public response time in Canada was longer, and the duration of public attention was shorter than that in the USA, the UK and Australia. In addition, since the first reported case of COVID-19 in Canada, presenting as mild pneumonia, which may, to some extent, reduce the public’s vigilance to this newly virus [26]. Besides, according to previous research in China, the average duration of netizens’ attention to social events is around 21 days [27]. Since the outbreak of COVID-19 meets the criteria for a PHEC, the duration of public attention toward ARS-CoV-2 should be longer. However, the duration of public attention in the countries contained in our study was not very long, particularly in Ireland and New Zealand. During the earlier period of observing time, all the countries contained in our study did not present too much attention to COVID-19, which may contribute to the global outbreak of COVID-19 in recent days. Which reminds us that when facing public health emergencies, governments should strengthen the publicity of these issues, shorten the response time to deal with major emergencies, improve the public's vigilance and sensitivity, inform public the importance of protecting themselves with enough precautionary measures, so as to minimize the loss of the masses.

Our results also suggest that RSV for “2019-nCoV + SARS-CoV-2 + novel coronavirus + new coronavirus + COVID-19 + Corona Virus Disease 2019” were slight positively correlated with the daily number of confirmed cases in both China and the total number of infected patients in other countries (except China) in most countries contained in our study. This indicates that with the case number soaring worldwide, people’s interest in COVID-19 did not increase that much. Interestingly, no correlation was observed between the RSV regarding COVID-19 in the USA and the total number of cases in other countries (except China). Which means the people in the USA did not care enough for COVID-19.

Besides, data retrieved from the USA, the UK, Canada, and Australia by Google Trends, remind us that people from different countries had a common interest in “coronavirus symptoms” as well. The outbreak of COVID-19 started in late December in winter, which is also the season when the viruses of influenza and respiratory syncytial circulation peaks [28]. Moreover, similar to the symptoms of influenza, the most common symptoms for patients confirmed with COVID-19 are fever, cough, and myalgia or fatigue, some have a headache, hemoptysis, and diarrhea [29]. So, most people searched the term “coronavirus symptoms” on Google, afraid of being infected with SARS-CoV-2. Interestingly, the most relative searched terms in the USA, the UK, Canada, and New Zealand were “coronavirus new york”, “coronavirus uk”, “coronavirus canada” and “new zealand coronavirus”, respectively, which may due to that people cared more about the epidemic of the COVID-19 in their own countries. Besides, people from Canada also had a great interest in the term regarding “lunar new year”. Since it has been discovered that COVID-19 can transmit from human to human [30], the Spring Festival travel rush during Chinese New Year can lead to further spread of this newly coronavirus. Thus, people in these countries might be worried that more people will be infected after the Spring Festival. Besides, the COVID-19 outbreak and spread speedily during the Spring Festival, which may also associate with our results.

Additionally, people from different sub-regions within each country pay different attention to the topic regarding COVID-19. According to the population statistics report from different countries, the areas with the largest distribution of Chinese were: New York, New Jersey in the United States [31]; England in the UK [32]; Ontario, British Columbia in Canada [33]; New South Wales, Victoria in Australia [34]; Auckland in New Zealand [35]. Interestingly, all the regions with the largest distribution of Chinese presented a higher interest to the topic regarding COVID-19, which suggests that the distribution of RSV for “2019-nCoV + SARS-CoV-2 + novel coronavirus + new coronavirus + COVID-19 + Corona Virus Disease 2019” may associate with the distribution of Chinese within regions. Moreover, our results also indicate that sub-regions in the UK, the USA, Canada and Australia with a higher awareness regarding COVID-19 are places with the developed economy, dense population and developed transportation system. On the contrary, the public in Northwest Territories, Nunavut, and Yukon, places in the northwest of Canada with a sparse population, did not present too much attention to the topic regarding SARS-CoV-2. Additionally, Otago, a sub-region with higher awareness regarding SARS-CoV-2 in New Zealand, is world-famous because of its tourist city- Queenstown. According to the WHO’s report that most people confirmed with COVID-19 in other countries had the history of traveling in Wuhan, China. Therefore, the development of tourism in different sub-regions may also affect the public awareness of COVID-19. Besides, according to a previous study’s report, as of 8 February 2020, the confirmed cases in Australia were found in New South Wales (4 cases), Victoria (4 cases), Queensland (5 cases) and Southern Australia (2 cases) [25]. The public in all the regions presented a higher interest in the topic regarding SARS-CoV-2. So, the confirmed cases within the regions may also affect our results. However, further studies are required to confirm these hypotheses. Since the distribution of the public with a higher awareness of COVID-19 was centralized, people in other places did not pay enough attention to COVID-19. Therefore, governments in all countries are required to strengthen the propaganda of COVID-19 nationally, so as to prevent the transmission of COVID-19 globally.

Several inherent limitations of our research should be acknowledged. First, we only use the single search engine, Google, to retrieve data from the main English-speaking countries from both hemispheres. Thus, selection bias will be existing as Google only gathering the data of particular groups of people who chose to get the information by using this search engine. Second, the detailed information about the procedures that Google implements to analyze this search behavior and the characteristics of the user who searched by Google remain unclear. Therefore, to control other factors which may affect our results would be impossible.

## CONCLUSIONS

In summary, a similar search trend for “2019-nCoV + SARS-CoV-2 + novel coronavirus + new coronavirus + COVID-19 + Corona Virus Disease 2019” was found in both Northern and Southern hemisphere with the first peak occurred around 31^st^ January 2020. People in the United States, the UK, and Australia responded faster toward COVID-19 due to the effective propaganda methods taken in these countries. Nevertheless, the duration of public attention in all the countries from both hemispheres contained in our study was not very long, especially in Ireland and New Zealand. The correlation between RSV related to COVID-19 with the daily number of confirmed cases in China and the total daily number of people infected with SARS-CoV-2 in other countries (besides China) was slight positively correlated. This suggests that public awareness of COVID-19 was not strong enough. Thus, more effective measures should be taken in these countries to strengthen the propaganda of COVID-19 to enhance public awareness and finally control the spread of COVID-19 worldwide.

## Additional material

Online Supplementary Document
