# Supplementary material for: More effective strategies are required to strengthen public awareness of COVID-19: Evidence from Google Trends
Source: J Glob Health. 2020 Apr 15;10(1):011003. doi: 10.7189/jogh.10.011003 (PMC7182392; doi:10.7189/jogh.10.011003)

Table S1. Dynamic series analysis of the search volumes for the topic regarding COVID-19 in Canada, Ireland, Australia, New Zealand, the Northern hemisphere and the Southern hemisphere

Countries/Hemispheres	Date	RSV	Absolute increment		development rate（%）		increment rate（%）	
			Cumulative	Day on day	Fixed base ratio	link relative ratio	Fixed base ratio	link relative ratio
Canada	13-Jan-20	2	_	_	100.0	100.0	_	_
	14-Jan-20	1	-1	-1	50.0	50.0	-50.0	-50.0
	15-Jan-20	1	-1	0	50.0	100.0	-50.0	0.0
	16-Jan-20	1	-1	0	50.0	100.0	-50.0	0.0
	17-Jan-20	2	0	1	100.0	200.0	0.0	100.0
	18-Jan-20	1	-1	-1	50.0	50.0	-50.0	-50.0
	19-Jan-20	3	1	2	150.0	300.0	50.0	200.0
	20-Jan-20	9	7	6	450.0	300.0	350.0	200.0
	21-Jan-20	23	21	14	1150.0	255.6	1050.0	155.6
	22-Jan-20	33	31	10	1650.0	143.5	1550.0	43.5
	23-Jan-20	39	37	6	1950.0	118.2	1850.0	18.2
	24-Jan-20	50	48	11	2500.0	128.2	2400.0	28.2
	25-Jan-20	100	98	50	5000.0	200.0	4900.0	100.0
	26-Jan-20	94	92	-6	4700.0	94.0	4600.0	-6.0
	27-Jan-20	73	71	-21	3650.0	77.7	3550.0	-22.3
	28-Jan-20	85	83	12	4250.0	116.4	4150.0	16.4
	29-Jan-20	81	79	-4	4050.0	95.3	3950.0	-4.7
	30-Jan-20	73	71	-8	3650.0	90.1	3550.0	-9.9
	31-Jan-20	72	70	-1	3600.0	98.6	3500.0	-1.4
	01-Feb-20	45	43	-27	2250.0	62.5	2150.0	-37.5
	02-Feb-20	46	44	1	2300.0	102.2	2200.0	2.2
	03-Feb-20	49	47	3	2450.0	106.5	2350.0	6.5
	04-Feb-20	51	49	2	2550.0	104.1	2450.0	4.1
	05-Feb-20	41	39	-10	2050.0	80.4	1950.0	-19.6
	06-Feb-20	34	32	-7	1700.0	82.9	1600.0	-17.1
	07-Feb-20	44	42	10	2200.0	129.4	2100.0	29.4
	08-Feb-20	39	37	-5	1950.0	88.6	1850.0	-11.4

	09-Feb-20	36	34	-3	1800.0	92.3	1700.0	-7.7
	10-Feb-20	38	36	2	1900.0	105.6	1800.0	5.6
	11-Feb-20	46	44	8	2300.0	121.1	2200.0	21.1
	12-Feb-20	78	76	32	3900.0	169.6	3800.0	69.6
	13-Feb-20	64	62	-14	3200.0	82.1	3100.0	-17.9
	14-Feb-20	43	41	-21	2150.0	67.2	2050.0	-32.8
	15-Feb-20	56	54	13	2800.0	130.2	2700.0	30.2
	16-Feb-20	58	56	2	2900.0	103.6	2800.0	3.6
	17-Feb-20	39	37	-19	1950.0	67.2	1850.0	-32.8
	18-Feb-20	49	47	10	2450.0	125.6	2350.0	25.6
	19-Feb-20	41	39	-8	2050.0	83.7	1950.0	-16.3
	20-Feb-20	45	43	4	2250.0	109.8	2150.0	9.8
	21-Feb-20	56	54	11	2800.0	124.4	2700.0	24.4
	22-Feb-20	36	34	-20	1800.0	64.3	1700.0	-35.7
	23-Feb-20	53	51	17	2650.0	147.2	2550.0	47.2
	24-Feb-20	96	94	43	4800.0	181.1	4700.0	81.1
Ireland	20-Jan-20	18	_	_	100.0	100.0	_	_
	21-Jan-20	26	8	8	144.4	144.4	44.4	44.4
	22-Jan-20	53	35	27	294.4	203.8	194.4	103.8
	23-Jan-20	26	8	-27	144.4	49.1	44.4	-50.9
	24-Jan-20	83	65	57	461.1	319.2	361.1	219.2
	25-Jan-20	79	61	-4	438.9	95.2	338.9	-4.8
	26-Jan-20	65	47	-14	361.1	82.3	261.1	-17.7
	27-Jan-20	60	42	-5	333.3	92.3	233.3	-7.7
	28-Jan-20	96	78	36	533.3	160.0	433.3	60.0
	29-Jan-20	71	53	-25	394.4	74.0	294.4	-26.0
	30-Jan-20	81	63	10	450.0	114.1	350.0	14.1
	31-Jan-20	100	82	19	555.6	123.5	455.6	23.5
	01-Feb-20	59	41	-41	327.8	59.0	227.8	-41.0
	02-Feb-20	75	57	16	416.7	127.1	316.7	27.1
	03-Feb-20	32	14	-43	177.8	42.7	77.8	-57.3
	04-Feb-20	53	35	21	294.4	165.6	194.4	65.6
	05-Feb-20	45	27	-8	250.0	84.9	150.0	-15.1
	06-Feb-20	37	19	-8	205.6	82.2	105.6	-17.8

	07-Feb-20	37	19	0	205.6	100.0	105.6	0.0
	08-Feb-20	38	20	1	211.1	102.7	111.1	2.7
	09-Feb-20	52	34	14	288.9	136.8	188.9	36.8
	10-Feb-20	33	15	-19	183.3	63.5	83.3	-36.5
	11-Feb-20	69	51	36	383.3	209.1	283.3	109.1
	12-Feb-20	72	54	3	400.0	104.3	300.0	4.3
	13-Feb-20	55	37	-17	305.6	76.4	205.6	-23.6
	14-Feb-20	76	58	21	422.2	138.2	322.2	38.2
	15-Feb-20	37	19	-39	205.6	48.7	105.6	-51.3
	16-Feb-20	47	29	10	261.1	127.0	161.1	27.0
	17-Feb-20	35	17	-12	194.4	74.5	94.4	-25.5
	18-Feb-20	43	25	8	238.9	122.9	138.9	22.9
	19-Feb-20	52	34	9	288.9	120.9	188.9	20.9
	20-Feb-20	18	0	-34	100.0	34.6	0.0	-65.4
	21-Feb-20	45	27	27	250.0	250.0	150.0	150.0
	22-Feb-20	78	60	33	433.3	173.3	333.3	73.3
	23-Feb-20	66	48	-12	366.7	84.6	266.7	-15.4
	24-Feb-20	77	59	11	427.8	116.7	327.8	16.7
Australia	09-Jan-20	2	_	_	100.0	100.0	_	_
	10-Jan-20	0	-2	-2	0.0	0.0	-100.0	-100.0
	11-Jan-20	0	-2	0	0.0	_	-100.0	_
	12-Jan-20	0	-2	0	0.0	_	-100.0	_
	13-Jan-20	2	0	2	100.0	_	0.0	_
	14-Jan-20	2	0	0	100.0	100.0	0.0	0.0
	15-Jan-20	0	-2	-2	0.0	0.0	-100.0	-100.0
	16-Jan-20	0	-2	0	0.0	_	-100.0	_
	17-Jan-20	0	-2	0	0.0	_	-100.0	_
	18-Jan-20	0	-2	0	0.0	_	-100.0	_
	19-Jan-20	3	1	3	150.0	_	50.0	_
	20-Jan-20	3	1	0	150.0	100.0	50.0	0.0
	21-Jan-20	23	21	20	1150.0	766.7	1050.0	666.7
	22-Jan-20	26	24	3	1300.0	113.0	1200.0	13.0
	23-Jan-20	25	23	-1	1250.0	96.2	1150.0	-3.8
	24-Jan-20	30	28	5	1500.0	120.0	1400.0	20.0

25-Jan-20	84	82	54	4200.0	280.0	4100.0	180.0
26-Jan-20	66	64	-18	3300.0	78.6	3200.0	-21.4
27-Jan-20	60	58	-6	3000.0	90.9	2900.0	-9.1
28-Jan-20	79	77	19	3950.0	131.7	3850.0	31.7
29-Jan-20	89	87	10	4450.0	112.7	4350.0	12.7
30-Jan-20	100	98	11	5000.0	112.4	4900.0	12.4
31-Jan-20	70	68	-30	3500.0	70.0	3400.0	-30.0
01-Feb-20	81	79	11	4050.0	115.7	3950.0	15.7
02-Feb-20	64	62	-17	3200.0	79.0	3100.0	-21.0
03-Feb-20	52	50	-12	2600.0	81.3	2500.0	-18.8
04-Feb-20	48	46	-4	2400.0	92.3	2300.0	-7.7
05-Feb-20	58	56	10	2900.0	120.8	2800.0	20.8
06-Feb-20	43	41	-15	2150.0	74.1	2050.0	-25.9
07-Feb-20	53	51	10	2650.0	123.3	2550.0	23.3
08-Feb-20	40	38	-13	2000.0	75.5	1900.0	-24.5
09-Feb-20	37	35	-3	1850.0	92.5	1750.0	-7.5
10-Feb-20	38	36	1	1900.0	102.7	1800.0	2.7
11-Feb-20	55	53	17	2750.0	144.7	2650.0	44.7
12-Feb-20	70	68	15	3500.0	127.3	3400.0	27.3
13-Feb-20	71	69	1	3550.0	101.4	3450.0	1.4
14-Feb-20	54	52	-17	2700.0	76.1	2600.0	-23.9
15-Feb-20	51	49	-3	2550.0	94.4	2450.0	-5.6
16-Feb-20	26	24	-25	1300.0	51.0	1200.0	-49.0
17-Feb-20	34	32	8	1700.0	130.8	1600.0	30.8
18-Feb-20	33	31	-1	1650.0	97.1	1550.0	-2.9
19-Feb-20	35	33	2	1750.0	106.1	1650.0	6.1
20-Feb-20	39	37	4	1950.0	111.4	1850.0	11.4
21-Feb-20	38	36	-1	1900.0	97.4	1800.0	-2.6
22-Feb-20	29	27	-9	1450.0	76.3	1350.0	-23.7

	23-Feb-20	55	53	26	2750.0	189.7	2650.0	89.7
	24-Feb-20	65	63	10	3250.0	118.2	3150.0	18.2
New Zealand	20-Jan-20	4	_	_	100.0	100.0	_	_
	21-Jan-20	10	6	6	250.0	250.0	150.0	150.0
	22-Jan-20	8	4	-2	200.0	80.0	100.0	-20.0
	23-Jan-20	24	20	16	600.0	300.0	500.0	200.0
	24-Jan-20	27	23	3	675.0	112.5	575.0	12.5
	25-Jan-20	65	61	38	1625.0	240.7	1525.0	140.7
	26-Jan-20	50	46	-15	1250.0	76.9	1150.0	-23.1
	27-Jan-20	45	41	-5	1125.0	90.0	1025.0	-10.0
	28-Jan-20	70	66	25	1750.0	155.6	1650.0	55.6
	29-Jan-20	60	56	-10	1500.0	85.7	1400.0	-14.3
	30-Jan-20	100	96	40	2500.0	166.7	2400.0	66.7
	31-Jan-20	90	86	-10	2250.0	90.0	2150.0	-10.0
	01-Feb-20	78	74	-12	1950.0	86.7	1850.0	-13.3
	02-Feb-20	69	65	-9	1725.0	88.5	1625.0	-11.5
	03-Feb-20	63	59	-6	1575.0	91.3	1475.0	-8.7
	04-Feb-20	44	40	-19	1100.0	69.8	1000.0	-30.2
	05-Feb-20	49	45	5	1225.0	111.4	1125.0	11.4
	06-Feb-20	50	46	1	1250.0	102.0	1150.0	2.0
	07-Feb-20	62	58	12	1550.0	124.0	1450.0	24.0
	08-Feb-20	46	42	-16	1150.0	74.2	1050.0	-25.8
	09-Feb-20	52	48	6	1300.0	113.0	1200.0	13.0
	10-Feb-20	70	66	18	1750.0	134.6	1650.0	34.6
	11-Feb-20	43	39	-27	1075.0	61.4	975.0	-38.6
	12-Feb-20	38	34	-5	950.0	88.4	850.0	-11.6
	13-Feb-20	42	38	4	1050.0	110.5	950.0	10.5
	14-Feb-20	60	56	18	1500.0	142.9	1400.0	42.9
	15-Feb-20	43	39	-17	1075.0	71.7	975.0	-28.3
	16-Feb-20	37	33	-6	925.0	86.0	825.0	-14.0
	17-Feb-20	35	31	-2	875.0	94.6	775.0	-5.4
	18-Feb-20	31	27	-4	775.0	88.6	675.0	-11.4
	19-Feb-20	20	16	-11	500.0	64.5	400.0	-35.5

	20-Feb-20	31	27	11	775.0	155.0	675.0	55.0
	21-Feb-20	28	24	-3	700.0	90.3	600.0	-9.7
	22-Feb-20	19	15	-9	475.0	67.9	375.0	-32.1
	23-Feb-20	29	25	10	725.0	152.6	625.0	52.6
	24-Feb-20	68	64	39	1700.0	234.5	1600.0	134.5
Northern hemisphere	02-Jan-20	1	_	_	100.0	100.0	_	_
	03-Jan-20	0	-1	-1	0.0	0.0	-100.0	-100.0
	04-Jan-20	0	-1	0	0.0	_	-100.0	_
	05-Jan-20	0	-1	0	0.0	_	-100.0	_
	06-Jan-20	0	-1	0	0.0	_	-100.0	_
	07-Jan-20	0	-1	0	0.0	_	-100.0	_
	08-Jan-20	1	0	1	100.0	_	0.0	_
	09-Jan-20	2	1	1	200.0	200.0	100.0	100.0
	10-Jan-20	2	1	0	200.0	100.0	100.0	0.0
	11-Jan-20	2	1	0	200.0	100.0	100.0	0.0
	12-Jan-20	1	0	-1	100.0	50.0	0.0	-50.0
	13-Jan-20	2	1	1	200.0	200.0	100.0	100.0
	14-Jan-20	3	2	1	300.0	150.0	200.0	50.0
	15-Jan-20	3	2	0	300.0	100.0	200.0	0.0
	16-Jan-20	3	2	0	300.0	100.0	200.0	0.0
	17-Jan-20	5	4	2	500.0	166.7	400.0	66.7
	18-Jan-20	6	5	1	600.0	120.0	500.0	20.0
	19-Jan-20	4	3	-2	400.0	66.7	300.0	-33.3
	20-Jan-20	45	44	41	4500.0	1125.0	4400.0	1025.0
	21-Jan-20	88	87	43	8800.0	195.6	8700.0	95.6
	22-Jan-20	139	138	51	13900.0	158.0	13800.0	58.0
	23-Jan-20	162	161	23	16200.0	116.5	16100.0	16.5
	24-Jan-20	275	274	113	27500.0	169.8	27400.0	69.8
	25-Jan-20	332	331	57	33200.0	120.7	33100.0	20.7
	26-Jan-20	281	280	-51	28100.0	84.6	28000.0	-15.4
	27-Jan-20	258	257	-23	25800.0	91.8	25700.0	-8.2
	28-Jan-20	341	340	83	34100.0	132.2	34000.0	32.2
	29-Jan-20	290	289	-51	29000.0	85.0	28900.0	-15.0

	30-Jan-20	322	321	32	32200.0	111.0	32100.0	11.0
	31-Jan-20	364	363	42	36400.0	113.0	36300.0	13.0
	01-Feb-20	246	245	-118	24600.0	67.6	24500.0	-32.4
	02-Feb-20	248	247	2	24800.0	100.8	24700.0	0.8
	03-Feb-20	207	206	-41	20700.0	83.5	20600.0	-16.5
	04-Feb-20	214	213	7	21400.0	103.4	21300.0	3.4
	05-Feb-20	184	183	-30	18400.0	86.0	18300.0	-14.0
	06-Feb-20	162	161	-22	16200.0	88.0	16100.0	-12.0
	07-Feb-20	191	190	29	19100.0	117.9	19000.0	17.9
	08-Feb-20	175	174	-16	17500.0	91.6	17400.0	-8.4
	09-Feb-20	180	179	5	18000.0	102.9	17900.0	2.9
	10-Feb-20	183	182	3	18300.0	101.7	18200.0	1.7
	11-Feb-20	248	247	65	24800.0	135.5	24700.0	35.5
	12-Feb-20	324	323	76	32400.0	130.6	32300.0	30.6
	13-Feb-20	261	260	-63	26100.0	80.6	26000.0	-19.4
	14-Feb-20	235	234	-26	23500.0	90.0	23400.0	-10.0
	15-Feb-20	175	174	-60	17500.0	74.5	17400.0	-25.5
	16-Feb-20	171	170	-4	17100.0	97.7	17000.0	-2.3
	17-Feb-20	159	158	-12	15900.0	93.0	15800.0	-7.0
	18-Feb-20	179	178	20	17900.0	112.6	17800.0	12.6
	19-Feb-20	168	167	-11	16800.0	93.9	16700.0	-6.1
	20-Feb-20	141	140	-27	14100.0	83.9	14000.0	-16.1
	21-Feb-20	184	183	43	18400.0	130.5	18300.0	30.5
	22-Feb-20	202	201	18	20200.0	109.8	20100.0	9.8
	23-Feb-20	219	218	17	21900.0	108.4	21800.0	8.4
	24-Feb-20	343	342	124	34300.0	156.6	34200.0	56.6
Southern hemisphere	09-Jan-20	2	_	_	100.0	100.0	_	_
	10-Jan-20	0	-2	-2	0.0	0.0	-100.0	-100.0
	11-Jan-20	0	-2	0	0.0	_	-100.0	_
	12-Jan-20	0	-2	0	0.0	_	-100.0	_
	13-Jan-20	2	0	2	100.0	_	0.0	_

14-Jan-20	2	0	0	100.0	100.0	0.0	0.0
15-Jan-20	0	-2	-2	0.0	0.0	-100.0	-100.0
16-Jan-20	0	-2	0	0.0	_	-100.0	_
17-Jan-20	0	-2	0	0.0	_	-100.0	_
18-Jan-20	0	-2	0	0.0	_	-100.0	_
19-Jan-20	3	1	3	150.0	_	50.0	_
20-Jan-20	7	5	4	350.0	233.3	250.0	133.3
21-Jan-20	33	31	26	1650.0	471.4	1550.0	371.4
22-Jan-20	34	32	1	1700.0	103.0	1600.0	3.0
23-Jan-20	49	47	15	2450.0	144.1	2350.0	44.1
24-Jan-20	57	55	8	2850.0	116.3	2750.0	16.3
25-Jan-20	149	147	92	7450.0	261.4	7350.0	161.4
26-Jan-20	116	114	-33	5800.0	77.9	5700.0	-22.1
27-Jan-20	105	103	-11	5250.0	90.5	5150.0	-9.5
28-Jan-20	149	147	44	7450.0	141.9	7350.0	41.9
29-Jan-20	149	147	0	7450.0	100.0	7350.0	0.0
30-Jan-20	200	198	51	10000.0	134.2	9900.0	34.2
31-Jan-20	160	158	-40	8000.0	80.0	7900.0	-20.0
01-Feb-20	159	157	-1	7950.0	99.4	7850.0	-0.6
02-Feb-20	133	131	-26	6650.0	83.6	6550.0	-16.4
03-Feb-20	115	113	-18	5750.0	86.5	5650.0	-13.5
04-Feb-20	92	90	-23	4600.0	80.0	4500.0	-20.0
05-Feb-20	107	105	15	5350.0	116.3	5250.0	16.3
06-Feb-20	93	91	-14	4650.0	86.9	4550.0	-13.1
07-Feb-20	115	113	22	5750.0	123.7	5650.0	23.7
08-Feb-20	86	84	-29	4300.0	74.8	4200.0	-25.2
09-Feb-20	89	87	3	4450.0	103.5	4350.0	3.5
10-Feb-20	108	106	19	5400.0	121.3	5300.0	21.3
11-Feb-20	98	96	-10	4900.0	90.7	4800.0	-9.3
12-Feb-20	108	106	10	5400.0	110.2	5300.0	10.2
13-Feb-20	113	111	5	5650.0	104.6	5550.0	4.6

Table S2. The correlation between RSV for the topic regarding COVID-19 and the number of confirmed cases

Index	Countries						Hemispheres	
	USA	UK	Canada	Ireland	Australia	New Zealand	Northern hemisphere	Southern hemisphere
14-Feb-20	114	112	1	5700.0	100.9	5600.0	0.9	
15-Feb-20	94	92	-20	4700.0	82.5	4600.0	-17.5	
16-Feb-20	63	61	-31	3150.0	67.0	3050.0	-33.0	
17-Feb-20	69	67	6	3450.0	109.5	3350.0	9.5	
18-Feb-20	64	62	-5	3200.0	92.8	3100.0	-7.2	
19-Feb-20	55	53	-9	2750.0	85.9	2650.0	-14.1	
20-Feb-20	70	68	15	3500.0	127.3	3400.0	27.3	
21-Feb-20	66	64	-4	3300.0	94.3	3200.0	-5.7	
22-Feb-20	48	46	-18	2400.0	72.7	2300.0	-27.3	
23-Feb-20	84	82	36	4200.0	175.0	4100.0	75.0	
24-Feb-20	133	131	49	6650.0	158.3	6550.0	58.3	

RSV: relative search volume

RSV in the Northern hemisphere was calculated by adding up the daily RSV in the USA, the UK, Canada and Ireland. Using the same way, we get the data in the Southern hemisphere by combining the daily RSV in Australia and New Zealand.

Confirmed cases in China	r_s	0.416	0.495	0.540	0.490	0.483	0.463	0.482	0.459
	p	0.004	$4.727x10^{-4}$	$1.090x10^{-4}$	0.001	0.001	0.001	0.001	0.001
Confirmed cases in other countries （except China）	r_s	0.289	0.388	0.439	0.380	0.372	0.348	0.368	0.343
	p	0.061	0.01	0.003	0.012	0.014	0.022	0.015	0.024

RSV：relative search volumes

Figure S1. Plots of time series for daily RSV of [2019-nCoV + SARS-CoV-2 + novel coronavirus + new coronavirus + COVID-19 + Corona Virus Disease 2019] and the daily number of confirmed cases with COVID-19 in China. RSV: relative search volume.

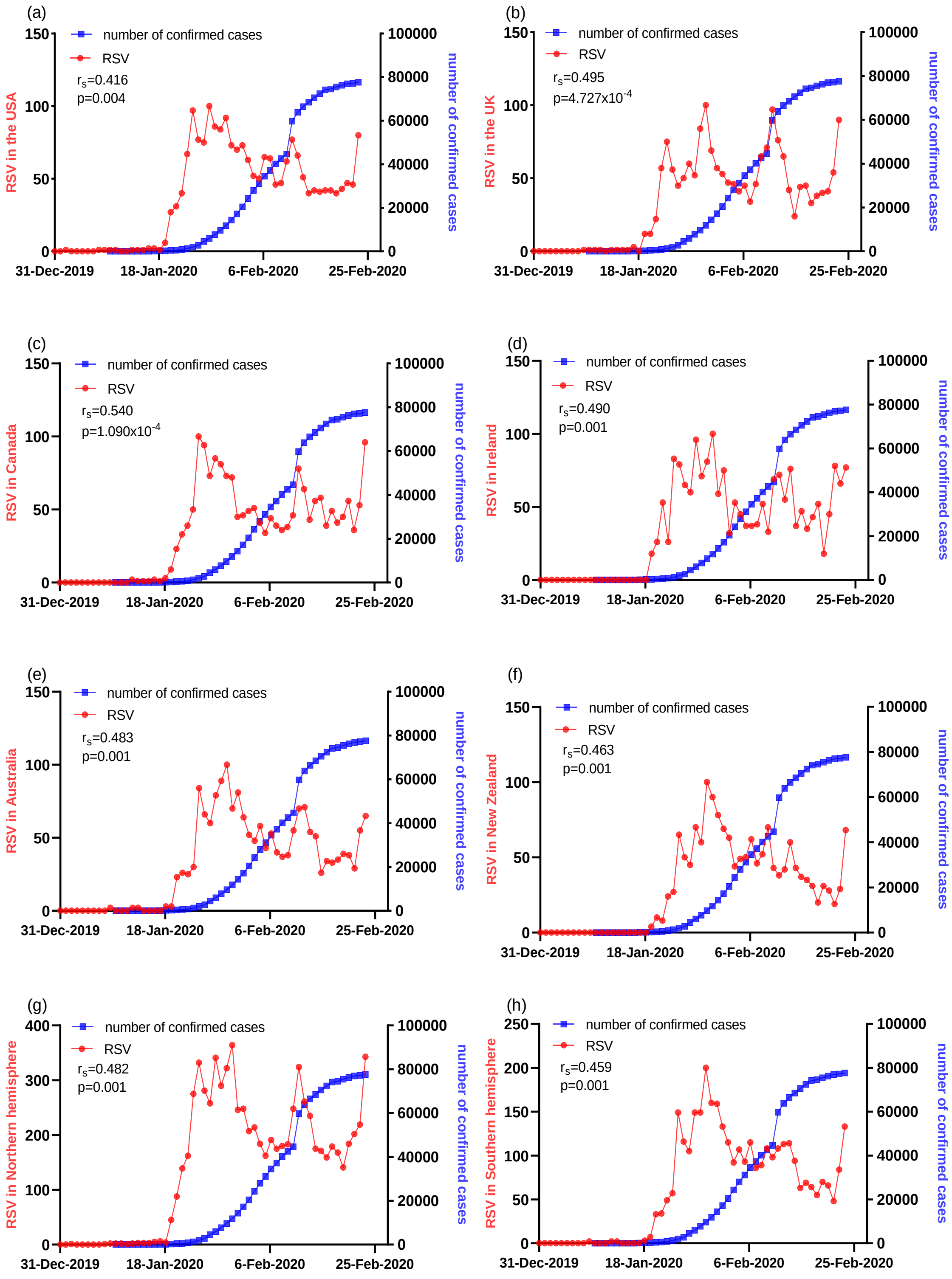

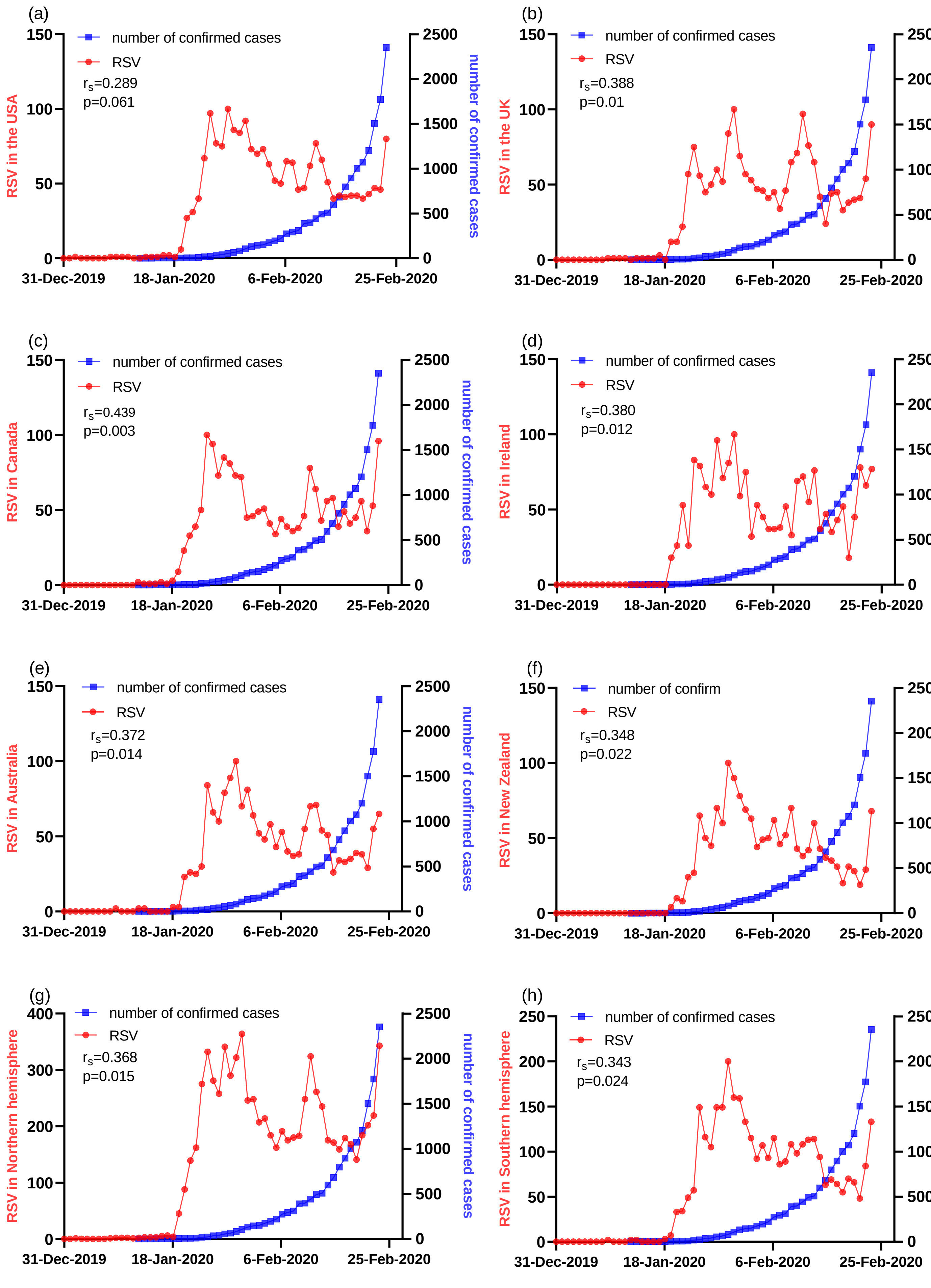
(a)
number of confirmed cases
RSV
r_s=0.289
p=0.061
RSV in the USA
number of confirmed cases
0
50
100
150
0
500
1000
1500
2000
2500
31-Dec-2019
18-Jan-2020
6-Feb-2020
25-Feb-2020
(b)
number of confirmed cases
RSV
r_s=0.388
p=0.01
RSV in the UK
(c)
number of confirmed cases
RSV
r_s=0.439
p=0.003
RSV in Canada
(d)
number of confirmed cases
RSV
r_s=0.380
p=0.012
RSV in Ireland
(e)
number of confirmed cases
RSV
r_s=0.372
p=0.014
RSV in Australia
(f)
number of confirm
RSV
r_s=0.348
p=0.022
RSV in New Zealand
(g)
number of confirmed cases
RSV
r_s=0.368
p=0.015
RSV in Northern hemisphere
0
100
200
300
400
(h)
number of confirmed cases
RSV
r_s=0.343
p=0.024
RSV in Southern hemisphere
0
50
100
150
200
250

Figure S2. Plots of time series for daily RSV of [2019-nCoV + SARS-CoV-2 + novel coronavirus + new coronavirus + COVID-19 + Corona Virus Disease 2019] and the total daily number of confirmed cases with COVID-19 in other countries (except China).

RSV: relative search volume.

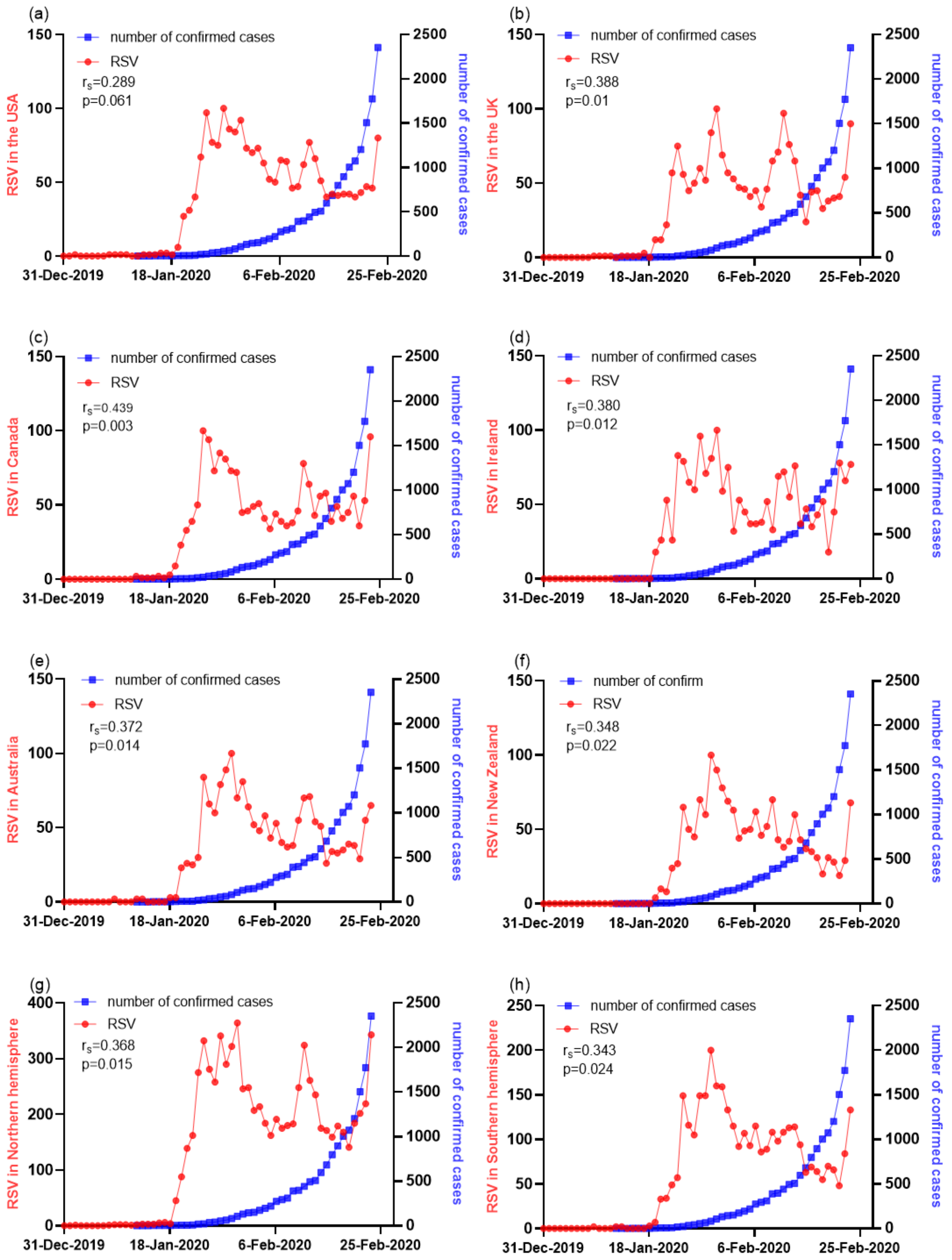

Supplement: Online Supplementary Document [file jogh-10-011003-s001.pdf]
